# Malnutrition and Diarrhœa in an Infant, from Improper Feeding

**Published:** 1879-04-20

**Authors:** 


### *MALNUTRITION AND DIARRHŒA IN AN INFANT, FROM IMPROPER FEEDING.*

BY DR. JACOBI.

The following history is given of this infant : Mary F., six weeks old, was healthy when born. When two weeks old the mother says that it had white spots in the mouth, which gradually increased in number. There were also spots on its legs, which looked like prickly heat. After that its bowels became loose, the evacuations being green in color and containing white lumps; and it began to lose flesh. Its buttocks, and genitals, and groins soon became sore and inflamed, and when it was a month old it commenced to vomit. It has also had a slight cough. The father and mother are healthy. They have had nine children, six of whom are still living. Of the three that died one was born very feeble and did not live long ; the second died of hooping-cough, and the third while teething, at the age of ten months. The mother has had but one miscarriage; and so we can, at all events, probably exclude syphilis as having anything to do with this infant's trouble. Neither does there seem to be any other hereditary taint, and, therefore, we must look for some cause or causes of the difficulty in the child itself.

The spots in the mouth, of which the mother spoke, were, no doubt, thrush, or *muguet*, as the French call it, and they have all disappeared; though the pharynx still presents a redder appearance than normal. On examination we find a well-marked erythema about the labia majora and arms, and also upon the inner surface of the thighs. An eruption of this character is usually due to external irritation, such as is liable to be set up by contact of fæces or urine with the skin, when perfect cleanliness is not observed. One of the most common of all the causes of infantile erythema is the use of diapers which have been wet with urine and then simply allowed to dry, without having first been washed. In the present instance the trouble is probably attributable to the diarrhœa from which the child has been suffering. As to the diarrhœa itself, the cause of that we shall no doubt find to have been improper feeding ; the food being either of an inappropriate kind, or else administered in too large quantities, or too frequently. The infant has also had another eruption, which has been described as prickly heat, and although prickly heat is not very often met with at this season of the year, I think the mother was probably right in so denominating it. It is easy enough to maintain summer heat in a bedroom, and the little vesicles seen in the eruption mentioned sometimes make their appearance on children who are kept too warm, and not allowed sufficient fresh air.

For the erythema I suggest oxide of zinc, or diachylon, ointment; or it may be that simply a powder will suffice. Licopodium, however, should never be used in such cases, as it unites with the effused serum to form a mass which is very irritating to the inflamed skin. The best powder here would probably be one composed of starch and oxide of zinc. To protect the parts most exposed to it from the irritation of the fæces and urine, a little cold-cream or simple cerate should be employed now and then. At the same time they should be kept as cool as possible.

As the diarrhœa, in all probability, depends upon improper feeding, it follows, of course, that in order to get rid of it we must address ourselves to the cause. The mother being unable to nurse the child at the breast, I would suggest that it should have cow's milk, which has been boiled and skimmed, diluted from one-half to one-third. Instead of adding simple water to the milk, however, we should employ a farinaceous gruel; and here barley water would be the most appropriate. Oatmeal, which would only tend to increase the diarrhœa in this case, is very useful when the bowels are constipated. If there is acidity a little lime water may be used. It is a great mistake, however, to suppose that lime water is really worth anything as an antacid, because so feeble is its action in this respect, that a vast quantity of it would in fact be necessary in order to produce any appreciable effect in neutralizing acidity by means of it. Still it is often very serviceable in such cases, and especially if there is but little acid present. It also has a very excellent effect in interfering to some extent with the coagulation of the milk; for it is found that when lime water is given with it the coagula are not so large or so firm as they would have been, had it not been employed. The white lumps in the child's passages, which were described by the mother, are made up of casein and fat, and they indicate either that the stomach does not digest properly, or that the quantity of milk taken by the child is too large. If necessary, the milk may be reduced until it is only in the proportion of one to three; and in case the diarrhœa should continue, it might perhaps be well to allow no milk at all for a period of twelve or twenty-four hours. In some cases it is desirable to withdraw the milk for even three or four days; in the meanwhile giving the child barley water and white of egg.—*Med. and Surg. Reporter.*

